# BMC Nursing reviewer acknowledgement, 2013

**DOI:** 10.1186/1472-6955-13-3

**Published:** 2014-02-04

**Authors:** Catia Cornacchia

**Affiliations:** 1 BioMed Central, Floor 6, 236 Gray's Inn Road, London, WC1X8HB, UK

## Abstract

**Contributing reviewers:**

The editors of BMC Nursing would like to thank all our reviewers who have contributed to the journal in Volume 12 (2013).

## 

Alison Kitson

Australia

Amanda Kenny

Australia

Ann Catrine Eldh

Sweden

Anneke Leijntje Francke

Netherlands

Ann-Kristin Karlsson

Sweden

Barbara Pieper

USA

Barbara Sittner

USA

Caitriona Cunningham

Ireland

Carina Berterö

Sweden

Carol Wong

Canada

Chidozie Mbada

Nigeria

Christine Brown Wilson

UK

Ciara Hughes

UK

Cornelia Heinze

Germany

David Stanley

Australia

Debra Dobbs

USA

Dimitri Beeckman

Belgium

Doris Howell

Canada

Dorothy Forbes

Canada

Edward Halloran

USA

Fiona Timmins

Ireland

Fiona Irvine

UK

Gerd Ahlstrom

Sweden

Gianluca Catania

Italy

Grace Lindsay

UK

Habiba Ali

United Arab Emirates

Heather Laschinger

Canada

Helen Hambly

UK

Helena Leino-Kilpi

Finland

Heng-Hsin Tung

Taiwan

Hiroshi Mikami

Japan

Ian Hodgson

UK

Ida Torunn Bjørk

Norway

James Balmford

Australia

Jane Phillips

Australia

Jane Conway

Australia

Joakim Ekberg

Sweden

Joanne Booth

UK

John Gregory

UK

Jose Egido

Spain

Joyce Wilkinson

UK

Juanita Hoe

UK

Julie Mytton

UK

Kaisa Haatainen

Finland

Karen Francis

Australia

Karen-Leigh Edward

Australia

Karin Zingmark

Sweden

Karli Treyvaud

Australia

Katherine Jones

USA

Kathleen Finlayson

Australia

Kim Jocelyn Usher

Australia

Kirsten Avlund

Denmark

Kristin Carson

Australia

Kuruvilla George

Australia

Leana Uys

South Africa

Lesley Wilkes

Australia

Lois Thomas

UK

Maarit Piirtola

Finland

Madawa Chandratilake

Sri Lanka

Mahvash Salsali

Iran

Marc Du Bois

Belgium

Marco Teli

Italy

Margaret Mcallister

Australia

Maria Müller-Staub

Switzerland

Marie Carney

Ireland

Mark Haddad

UK

Martin Mcnamara

Ireland

Michael Robling

UK

Min Hae Park

UK

Mohsen Adib-Hajbaghery

Iran

Nancy Hogan

USA

Neena Shah More

India

Nigel Wellman

UK

Nuhad Dumit

Lebanon

Oluwole Omolase

Nigeria

Patriek Mistiaen

Netherlands

Peter Gray

Australia

Peter Van Bogaert

Belgium

Peter Hj Van Der Voort

Netherlands

Pierre Cardinal

Canada

Pieter Van Den Hombergh

Netherlands

Rebecca P Winsett

USA

Renee Du Toit

South Africa

Rhian Parker

Australia

Robert Olson

Canada

Robyn Cant

Australia

Ruud Halfens

Netherlands

Salvatore R Maddi

USA

Shuh-Jen Sheu

Taiwan

Simon Cooper

Australia

Souraya Sidani

Canada

Steen Rosthoej

Denmark

Stefan Sävenstedt

Sweden

Steffen Fleischer

Germany

Steve Hemingway

UK

Suh-Ing Hsieh

Taiwan

Susan Broekmans

Belgium

Susan Barnason

USA

Sylvia Reitmanova

Canada

Tarja Suominen

Finland

Ulrike Dapp

Germany

Valerie Lapierre

France

Virpi Susanna Hantikainen

Switzerland

Zena Moore

Ireland

## Pre-publication history

The pre-publication history for this paper can be accessed here:

http://www.biomedcentral.com/1472-6955/13/3/prepub

